# Chemical Composition, Larvicidal Activity, and Enzyme Inhibition of the Essential Oil of *Lippia grata* Schauer from the Caatinga Biome against Dengue Vectors

**DOI:** 10.3390/ph14030250

**Published:** 2021-03-10

**Authors:** Stênio Freitas Felix, Alzeir Machado Rodrigues, Ana Livya Moreira Rodrigues, José Claudio Carneiro de Freitas, Daniela Ribeiro Alves, Alice Araújo da Silva, Dayanne Lima dos Santos, Kethelly Rayne Lima de Oliveira, Renato Almeida Montes, Marcus Vinicius Ferreira da Silva, Francisco Flávio da Silva Lopes, Selene Maia de Morais

**Affiliations:** 1 Departamento de Ensino, Instituto Federal de Educação, Ciência e Tecnologia do Ceará (IFCE), Campus Iguatu, Rodovia Iguatu/Várzea Alegre, km 05, s/n, Vila Cajazeiras, Iguatu, 63503-790 Ceará, Brazil; 2 Programa de Pós-Graduação em Biotecnologia, RENORBIO, Universidade Estadual do Ceará, Avenida Doutor Silas Munguba, 1700, Fortaleza, 60741-000 Ceará, Brazil; livya_rodrigues@hotmail.com; 3 Departamento de Ensino, Instituto Federal de Educação, Ciência e Tecnologia do Ceará (IFCE), Campus Acopiara/Rodovia CE 060, km 332, s/n, Vila Martins, Acopiara, 63560-000 Ceará, Brazil; alzeir.rodrigues@ifce.edu.br; 4 Laboratório de Análises Cromatográficas e Espectroscópicas, Universidade Estadual do Ceará, Avenida Doutor Silas Munguba, 1700, Fortaleza, 60741-000 Ceará, Brazil; flaviollopez@gmail.com; 5 Vertrauen Diagnosis, Avenida Washington Soares, 655, Fortaleza, 60810-000 Ceará, Brazil; joseclaudiocarneiro@yahoo.com; 6 Programa de Pós-Graduação em Ciências Naturais, Universidade Estadual do Ceará, Avenida Doutor Silas Munguba, 1700, Fortaleza, 60741-000 Ceará, Brazil; alves.danielaribeiro@gmail.com; 7 Laboratório de Química de Produtos Naturais, Universidade Estadual do Ceará, Avenida Doutor Silas Munguba, 1700, Fortaleza, 60741-000 Ceará, Brazil; alice.silva@aluno.uece.br (A.A.d.S.); dayanne.lima@aluno.uece.br (D.L.d.S.); kethellyrayne44@gmail.com (K.R.L.d.O.); renato_almeida18@outlook.com (R.A.M.); marcusviniciusuece@bol.com.br (M.V.F.d.S.); 8 Departamento de Química, Universidade Estadual do Ceará, Avenida Doutor Silas Munguba, 1700, Fortaleza, 60741-000 Ceará, Brazil

**Keywords:** *Aedes*, dengue, acetylcholinesterase, *Lippia*

## Abstract

Insect resistance and environmental pollution are among the drawbacks of continuous use of synthetic insecticides against the vectors of dengue, *Aedes* *aegypti* and *Aedes albopictus*. The objective of this study was to analyze the composition of the essential oil of *Lippia grata* Schauer collected from plants, in three periods of the year, to compare the larvicidal activity and enzymatic inhibition of the dengue vectors. The oilsanalyzed by gas chromatography coupled to mass spectrometry (GC-MS), presented thymol and 1,8-cineole, as the main constituents, in all three periods. This composition was different from that found in previous studies of the species from different places, thus, suggesting a new chemotype of *Lippia grata*. Larvicidal tests were performed at concentrations of 100, 75, 50, 25, and 12.5 μg.mL^−1^ and the essential oil from the rainy season showed the best results, with LC_50_ of 22.79 μg.mL^−1^ and 35.36 μg.mL^−1^ against *Ae*. *aegypti* and *Ae*. *albopictus*, respectively; this result was better than other reports. In the rainy period, however, there was a greater variety of components, which led to a better larvicidal effect, possibly due to synergistic action with minor constituents. Total proteins, amylases, and acetylcholinesterase of both species were inhibited by the oils.

## 1. Introduction

Dengue is a disease that causes thousands of deaths worldwide every year. The mosquito species *Ae*des *aegypti* and *Ae*des *albopictus* attract the interest of health officials and researchers around the world for being vectors of the dengue, yellow fever, chikungunya, and zika viruses, which cause diseases that affect all social classes, in turn causing economic losses, sequelae, and deaths, especially in tropical and subtropical countries [1,2]. The risks to the population occur due to human distribution in cities and suitable climate for mosquito reproduction, hampering attempts to control viruses [3].

In Brazil, dengue is reported in all regions of the country, with an increasing rate of infection and deaths. In 2020, until November 14, 971,136 dengue cases were registered, with an incidence rate of 462.1 cases per 100 thousand inhabitants, including 528 deaths confirmed in this period [4]. To control vectors, in addition to educational actions, it is necessary to discover effective products that reduce the population of vectors.

The use of synthetic chemicals has not been sufficiently effective in controlling *Aedes* spp., largely because of selective pressure for the development of resistant mosquito strains. However, bioprospecting for natural products can be a viable alternative to break the vector cycle without promoting resistance to conventional insecticides [5,6,7,8]. 

Insects have different mechanisms for resisting insecticides, promoted by enzymes capable of metabolizing substances that are toxic to the organism (xenobiotics) into nontoxic or rapidly excreted substances [9,10]. The use of synthetic insecticides together with natural products can promote changes (increasing or decreasing) in the levels of enzymes such as esterases, phosphatases, proteases, total proteins, amylases, and acetylcholinesterase, causing toxicity, resistance or processes that promote physiological dysfunctions resulting in death of the insect [10,11,12,13].

Essential oils synthesized by secondary plant metabolism have shown larvicidal potential, and their chemical constituents are capable of repelling insects, inhibiting enzymes, and causing deformities and mortality in various insect larval stages [12,14].

The genus *Lippia,* belonging to the family Verbenaceae, has approximately 200 species dispersed in South and Central America and Tropical Africa. In Brazil, 120 species are distributed in the Cerrado and Caatinga biomes [15]. Species such as *Lippia sidoides* [16,17,18], *Lippia mycrophylla* [19], *Lippia alba,* and *Lippia origanoides* [20] have shown larvicidal activity.

The species *Lippia grata* Schauer, popularly known as ”alecrim do mato” (“forest rosemary”), is endemic to Northeastern Brazil [21] and its essential oil has shown promising larvicidal activity. Studies of the larvicidal activity of essential oils of *L. grata* have been carried out in the states where the species is endemic. Different chemotypes tested against the species *Ae*. *aegypti* have shown different medium lethal concentrations (LC_50_), probably influenced by the action of the major constituents and synergism among the constituents [19,22,23]. Research on the larvicidal activity of essential oils against *Ae*. *albopictus* larvae is scarce, given the vector’s potential to spread arboviruses [24]. Other studies have identified that the essential oil yield, number of constituents, and relative proportions of secondary metabolites in plants are regulated by biotic and abiotic factors, such as season, area, and sample collection time, showing the relevance of chemical and biochemical studies with plants collected in different regions and at different times of the year [23,25].

The vegetation in Northeast Brazil ranges from the Atlantic Forest on the coast to the Mata dos Cocais in the middle north, with ecosystems such as mangroves, shrubland (Caatinga), savannah (Cerrado), and sandbank (Restinga), among others, which have exuberant fauna and flora, several endemic species, and endangered animals. Occupying almost 10% of the nation’s territory, with 844,453 km^2^, the Caatinga biome covers the states of Ceará, Rio Grande do Norte, Paraíba, Pernambuco, Sergipe, Alagoas, Bahia, south and east of Piauí, and north of Minas Gerais. In the Caatinga, there are approximately 5311 species of plants, of which at least 1547 are endemic [26]. Thus, there is a diversity of environments that guarantee a wide variety of plants with unique chemical constituents. This study evaluates the composition of the essential oils of *L*. *grata* collected in the Caatinga biome during the rainy (March), flowering (June), and dry (September) periods, as well as compares the larvicidal activity of the essential oils and enzymatic inhibition against the dengue vectors (*Ae*. *aegypti* and *Ae*. *albopictus*).

## 2. Results and Discussion 

### 2.1. Chemical Analysis of Lippia grata Essential Oils

The chemical composition of the essential oil of the leaves of *L*. *grata* was determined by GC–MSusing the Kovats index (KI) and comparing the mass spectra of each constituent with data from the literature [27].

The concentrations of essential oils from the rainy (REO), flowering (FEO), and dry (DEO) periods were 1.7%, 2.72%, and 1.2%, respectively. A previous study, conducted in the city of Crato, Ceará, obtained a yield 0.6%, lower than found in this study, from fresh leaves collected in the rainy season, whereas other studies with dried leaves of *L. grata* found similar yields to this study (1.9%, 2.6%, and 2.8%) [19,25,28]. 

**Figure 1:**
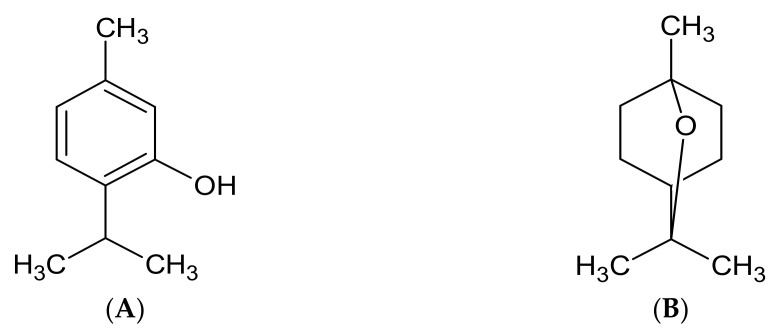
Structure of the major constituents of essential oils of *Lippia grata*. (**A**) Thymol; (**B**) 1,8-Cineole.

The essential oils of *L*. *grata* from other regions contain limonene, β-cariophyllene, o-cymene, camphor, linalool, α-pinene, thymol, and carvacrol [15]. The chemical analysis showed the constituents thymol and 1,8-cineole as main components during the three periods of the year, with the following yields: 58.46% and 9.43% (REO), 65.82% and 7% (FEO); and 73.49% and 13.58% (DEO) (Figure 1A,B). Quantified together, thymol and 1,8-cineole, two oxygenated monoterpenes, had yields of 67.89% (REO), 72.82% (FEO), and 87.07% (DEO). Therefore, in the dry season these constituents are present in higher concentrations. The chemical constituents carvacrol and *p*-cymene are described in several studies as major constituents; nevertheless, in this species, they were not identified or had low contents [29,30,31].

The chemical constituents in the three periods showed small variations in concentrations. The oil from plants collected in the rainy period had the greatest diversity, with 21 chemical constituents (Table 1). 

Previous studies of the essential oil of *L. grata* in the Northeast region of Brazil have shown the existence of several chemotypes (Table 2). Thus, the results of the chemical composition of the essential oil, in the present work, suggest the presence of another chemotype of *L. grata*, with a predominance of thymol and 1,8-cineole in the Caatinga biome.

According to Botrel et al. [36], the chemical composition and essential oil content of aromatic plants are influenced by atmospheric temperature and precipitation. According to Gobbo-Neto and Lopes [37], the time of collection of essential oils can also affect the composition, since the quantity and nature of the active constituents are variable during the year. Bitu et al. [25] suggested that the yield of the essential oil of *L. grata* is not greatly affected by the time of collection, but the use of fresh or dry leaves has a significant influence. Our results suggest that seasonality influences the concentration of chemical constituents and the yield of essential oil of *L. grata*, however, the presence of the main constituents remains unchanged. 

### 2.2. Larvicidal Activity of L. grata Essential Oils against Larvae of Ae. aegypti and Ae. albopictus

Considering the medium lethal concentration (LC_50_), the essential oil from rainy season, showed the best activity against both mosquitoes’ larvae. Regarding the LC_90_ against *Ae*. *aegypti* larvae, the REO also had the lowest concentration, i.e., 51.14 μg.mL^−1^ (Table 3). For LC_90_ against *Ae*. *albopictus*, the REO and DEO did not show significant differences in the concentration values.

In Brazil, studies of the larvicidal activity of the essential oil of *L*. *grata* carried out in the states of Maranhão, Sergipe, and Ceará have shown different major constituents and larvicidal activities. In Maranhão, the main constituents were 1,8-cineole and α-terpineol, with LC_50_ of 282 μg.mL^−1^ [23], and in Sergipe, carvacrol and *o*-cimene, with CL_50_ of 98 μg.mL^−1^ [22]. In the Ceará State, the main constituent was thymol with a LC_50_ of 26.3 μg.mL^−1^ against *Ae*. *aegypti* [19], which is a similar value to the present study. 

Previous studies with other species of the genus *Lippia* have shown LC_50_ values against *Ae*. *aegypti* higher than those demonstrated in the present study as follows: *Lippia sidoides* with CL_50_ of 63 μg.mL^−1^ and 56 μg.mL^−1^ [5,16]; *Lippia microphylla* with CL_50_ of 75.9 μg.mL^−1^, and *Lippia nodiflora* with CL_50_ of 107, 1 μg.mL^−1^ [19]; *Lippia origanoides* Kunth with CL_50_ of 187.3 μg.mL^−1^ [38]; and *Lippia alba* Mill with CL_50_ of 635.29 μg.mL^−1^ [39]. Therefore, the chemotype of *L*. *grata* from the Caatinga biome was more active than the other *Lippia* species, from other places.

To a lesser extent, there are studies about the activity of essential oils against *Ae*. *albopictus* larvae. *Croton nepetaefolius* presented the LC_50_ of 76.1 μg.mL^−1^ [24], *Allium macrostemon* showed LC_50_ of 72.86 μg.mL^−1^ [40], *Piper capitarianum* had LC_50_ of 161.81 μg.mL^−1^ [41];and *Rosmarinus officinalis* had LC_50_ of 104.6 μg.mL^−1^ [42].

The main constituents (thymol and 1,8-cineole) showed in previous studies larvicidal activity when tested individually against *Ae*. *aegypti* larvae, with LC_50_ values of 51.3 μg.mL^−1^ (thymol) and 47.9 μg.mL^−1^ (1,8-cineole), [19]. Thymol and 1,8-cineole tested against *Ae*. *albopictus* showed LC_50_ values of 12.9 μg.mL^−1^ and 73.5 μg.mL^−1^, respectively [42,43].

Synergism of the major chemical constituents in essential oils enhances the larvicidal action [22,44]. Thus, the REO, with the greatest diversity of constituents, had significantly different LC_50_ values than the other oils, with better larvicidal potential against *Ae*. *aegypti*. The LC_50_ values against *Ae*. *albopictus* of most essential oils were higher in relation to *Ae*. aegypti, except for the LC_50_ values of REO (*Ae*. *albopictus*) and DEO (*Ae*. *aegypti*), which showed no significant difference in the Tukey test. According to Rodrigues et al. [24], intra- and interspecific differences, due to different selective environmental pressures, can influence the resistance versus susceptibility of vectors.

### 2.3. Activity of the Essential Oils of L. grata on Enzymes of the Larvae of Ae. aegypti and Ae. albopictus

Studies of essential oils as larvicides have found biochemical alterations in the enzymes of *Aedes* spp. larvae. Enzymes such as phosphatases (alkaline and acidic), esterases, cytochrome 450, glutathione S-transferase, amylases, and acetylcholinesterase in insects perform functions of detoxification of xenobiotics and nutrition and are present mainly in larvae [45,46,47]. Alterations in the metabolism of these enzymes in insects can result in the capacity for resistance to insecticides when increased but death when inhibited or reduced, since they are responsible for detoxification and digestion, and hence are fundamental to the physiology of insects [13,48,49].

**Figure 2:**
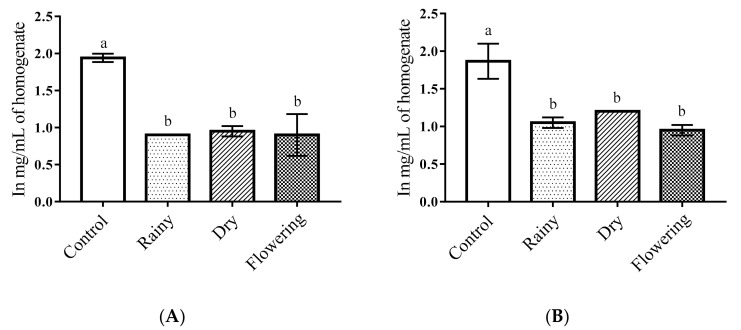
Biochemical changes in total proteins of *Ae*. *aegypti* and *Ae*. *albopictus* larvae. Changes in total protein levels between *Ae*. *aegypti* (**A**) and *Ae*. *albopictus* (**B**) larvae treated and not treated with the essential oils of *Lippia grata* Schauer. Different lowercase letters in the bar denote significant difference (*p* < 0.05). Bars represent standard deviations (*n* = 3). Values estimated by one-way ANOVA followed by the Tukey test. Control, untreated larvae.

The one-way ANOVA of the essential oils of *Lippia grata* Schauer showed a significant reduction in the total protein levels of the *Ae*. *aegypti* larvae as compared with the control (*p* < 0.05). The Tukey test showed there were no differences between the larvae treated with the REO, DEO, and FEO for total protein levels, but all treated larvae had significantly different levels than the control larvae. Similar results were observed for *Ae*. *albopictus* larvae (Figure 2A,B).

Changes in total proteins in insect larvae treated with plant extracts and essential oils have been identified in several studies [13,46,50]. *Annona muricata* extracts and formulations of the essential oils of *Azadira indica*, *Pongamia glabra,* and its extracts reduced the levels of total proteins and caused sluggishness, convulsions, paralysis, and death of *Ae*. *aegypti* and *Ae*. *albopictus* larvae [13,51]. Another study demonstrated an increase in total proteins after exposure of *Ae*. *aegypti* larvae to the extract of *Ziziphus jujube*, suggesting the production of detoxifying enzymes or defense proteins [52]. In the present study, we found a reduction in total proteins in both species when treated with essential oils. Such changes in the metabolism of total proteins lead to physiological changes, causing death of the larvae [13]. 

**Figure 3:**
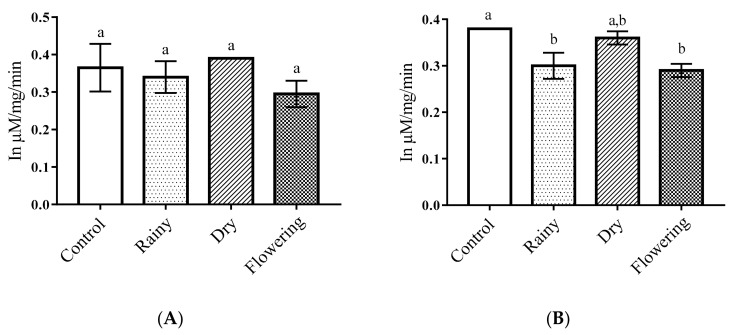
Biochemical changes in alkaline phosphatases of *Ae*. *aegypti* and *Ae*. *albopictus* larvae. Changes in alkaline phosphatases levels between *Ae*. *aegypti* (**A**) and *Ae*. *albopictus* (**B**) larvae treated and not treated with the essential oils of *Lippia grata* Schauer. Different lowercase letters in the bar denote significant difference (*p* < 0.05). Bars represent standard deviations (*n* = 3). Values estimated by one-way ANOVA followed by the Tukey test. Control, untreated larvae.

The essential oils did not alter the activity of alkaline phosphatase in *Ae*. *aegypti* larvae in relation to the control. The Tukey test did not indicate significant differences between larvae treated with the oils extracted from the leaves collected in the different periods as compared with the control. Regarding *Ae*. *albopictus*, there was a significant reduction in the amount of alkaline phosphatase in larvae treated with REO and FEO in terms of control (*p* < 0.05), and the Tukey test showed no differences between essential oils, while the larvae treated with DEO were similar to the control. Only *Ae*. *albopictus* larvae presented reduced enzyme level when treated REO and FEO (Figure 3A,B).

**Figure 4:**
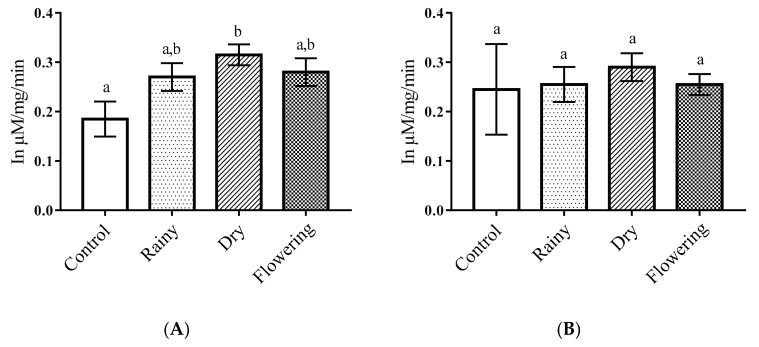
Biochemical changes in acid phosphatases of *Ae*. *aegypti* and *Ae*. *albopictus* larvae. Changes in acid phosphatases levels between *Ae*. *aegypti* (**A**) and *Ae*. *albopictus* (**B**) larvae treated and not treated with the essential oils of *Lippia grata*. Different lowercase letters in the bar denote significant difference (*p* < 0.05). Bars represent standard deviations (*n* = 3). Values estimated by one-way ANOVA followed by the Tukey test. Control, untreated larvae.

The levels of acid phosphatase in *Ae*. *aegypti* larvae treated with the DEO showed a significant increase of enzyme levels in relation to the control (*p* < 0.05). There were no differences between the essential oils, but the DEO showed a significant difference in relation to the control according to the Tukey test. *Ae*. *albopictus* larvae treated with essential oils had no significant reduction in relation to the control larvae (*p* < 0.05) and the Tukey test showed no differences between the larvae treated with the oils in relation to the treated samples and the control. Thus, only enzymes from *Ae*. *aegypti* larvae showed changes when treated with DEO (Figure 4A,B).

Alkaline and acid phosphatases can act as defensive enzymes in the body by detoxification of insecticides and ingested fatty structures. However, changes in metabolism caused by botanical biocides can interrupt physiological processes, causing death [46,53]. Studies with extracts of *Sapindus emarginatus* against *Ae*. *aegypti* larvae have shown a reduction in the levels of acid phosphatase, while there were no changes in the enzyme alkaline phosphatase, suggesting differential responses of the enzymes and impairment of the larval metabolism caused by the extract [53]. Thus, the significant increase in acid phosphatase in *Ae*. *aegypti* larvae when treated with the DEO possibly resulted from stimulation of the insects’ allelochemical defense mechanisms, while the significant reduction in alkaline phosphatases in *Ae*. *albopictus* larvae when treated with REO and FEO may be correlated with larval death due to enzymatic alteration and consequent dysfunction of physiology, suggesting different mechanisms of action of enzymes in the vectors and activity of chemical constituents present in the oils from the plants collected in the three periods studied. 

Factors such as harvest time, climate, soil characteristics, geographic origin, temperature, and the presence of flowers influence the biosynthesis of chemotypes and yield of essential oils, which can affect biological activity [54,55]. Thus, the action of essential oils on the enzymes, alkaline phosphatase and acid phosphatase, inhibiting or promoting their production, occurred either by the presence of various chemotypes present in essential oils that acted in synergism or through mechanisms of resistance to plant bioinsecticides. 

**Figure 5:**
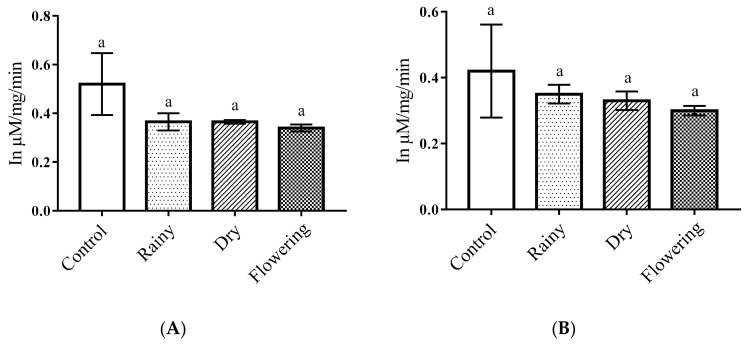
Biochemical changes in proteases of *Ae*. *aegypti* and *Ae*. *albopictus* larvae. Changes in protease levels between *Ae*. *aegypti* (**A**) and *Ae*. *albopictus* (**B**) larvae treated and not treated with the essential oils of *Lippia grata*. Different lowercase letters in the bar denote significant difference (*p* < 0.05). Bars represent standard deviations (*n* = 3). Values estimated by one-way ANOVA followed by the Tukey test. Control, untreated larvae.

The one-way ANOVA of the data on essential oils did not show significant reductions in proteases in the *Ae*. *aegypti* and *Ae*. *albopictus* larvae in relation to the control (*p* <. 0.05). The Tukey test showed no differences in protease levels in the larvae, and all samples treated were similar to the control (Figure 5A,B).

In insects, proteases break the peptide bonds of dietary proteins in the gut and can promote larval resistance to various toxins present in insecticides. However, this mechanism of action is possibly influenced by the larval stage [56,57,58]. Studies have shown that the death of *Aedes* spp. larvae is associated with the presence of protease inhibitors contained in plants, having defensive function, which act on the larval development by forming stable complexes with the intestinal proteases, inhibiting the hydrolytic activity of digestive enzymes [59,60]. Other studies have shown that *Moringa oleifera* lectins and *Lonchocarpus sericeus* proteins promote changes in levels of proteases in the intestine and cause death of *Ae*. *aegypti* larvae [49,61]. In the present study, the oils tested did not show significant influence against the proteases of the species, possibly due to the larval stage tested or the absence of inhibitors present in the oils.

**Figure 6:**
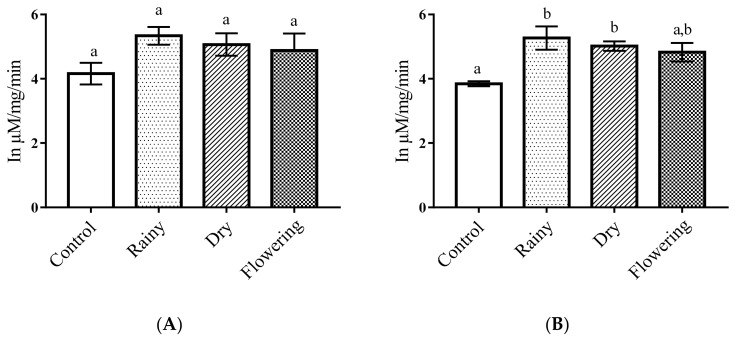
Biochemical changes in esterases of *Ae*. *aegypti* and *Ae*. *albopictus* larvae. Changes in esterase levels between *Ae*. *aegypti* (**A**) and *Ae*. *albopictus* (**B**) larvae treated and not treated with the essential oils of *Lippia grata*. Different lowercase letters in the bar denote significant difference (*p* < 0.05). Bars represent standard deviations (*n* = 3). Values estimated by one-way ANOVA followed by the Tukey test. Control, untreated larvae.

The essential oils did not cause significant reductions of esterases in the *Ae*. *aegypti* larvae (*p* < 0.05), and the Tukey test showed no differences between the larvae treated with the oils in relation to the control. As for *Ae*. *albopictus*, there was a significant increase in the enzyme activity in the larvae treated with REO and DEO in relation to the control (*p* < 0.05). The esterases of larvae treated with essential oils showed no differences, but in relation to the control, the esterases of the larvae treated with REO and DEO were different from the control, according to the Tukey test. When comparing the changes in the enzymes of the species, only enzymes from the larvae of *Ae*. *albopictus* showed changes in the levels of esterases (Figure 6A,B).

Insect esterases are important for the development of resistance to insecticides. They are widely distributed among insects, acting mainly on the metabolism of endogenous and exogenous substances, in addition to catalyzing the ester bonds present in most insecticides [13,62,63]. Some mechanisms may have stimulated the production of esterases in *Ae*. *albopictus* larvae exposed to REO and DEO. Different concentrations and diversity of the chemical constituents present in essential oils can influence the activity of esterases. Low concentrations can stimulate the expression of esterases, increasing their capacity for metabolization and detoxification, while high concentrations can produce toxic effects by inhibiting their activities [46,63].

Other factors that can influence the level of esterases are stages of development of the larvae, species, susceptibility and resistance of the insect, which can interfere in the level of esterases in treated larvae [46,56,64]. The essential oil of *Arisaema fargesii* inhibited the esterase activity of *Ae*. *aegypti* and *Ae*. *albopictus* larvae [65]. Studies of different species have shown that thymol promoted toxicity in *Plutella xylostella* larvae, causing an increase in esterases levels, whereas in *Ephestia kuehniella* Zeller, inhibition of these enzymes was observed [66,67]. Since all the essential oils had thymol as a major constituent, possibly one or more other constituents acted synergistically in REO and DEO, promoting an increase in the level of esterase, whereas FEO showed no significant difference in the levels of the enzyme in relation to the control. 

**Figure 7:**
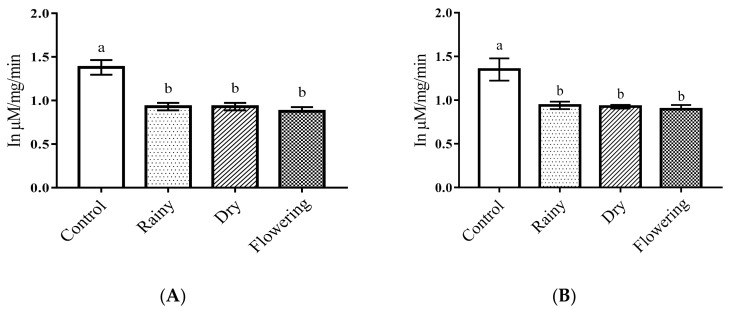
Biochemical changes in amylases of *Ae*. *aegypti* and *Ae*. *albopictus* larvae. Changes in amylase levels between *Ae*. *aegypti* (**A**) and *Ae*. *albopictus* (**B**) larvae treated and not treated with the essential oils of *Lippia grata*. Different lowercase letters in the bar denote significant difference (*p* < 0.05). Bars represent standard deviations (*n* = 3). Values estimated by one-way ANOVA followed by the Tukey test. Control, untreated larvae.

The amylase levels of *Ae*. *aegypti* and *Ae*. *albopictus* larvae treated with the essential oils showed significant reductions (*p* < 0.05). As indicated by the Tukey test, there was no difference between the larvae treated with the oils from the leaves collected in the three periods, but all the treated samples were significantly different from the control in the two species (Figure 7A,B).

Amylases are enzymes found in the midgut of insects that act in the hydrolysis of starch into maltose and glycogen into glucose, playing a fundamental role in the larval development of insects [11,67,68]. Studies with essential oils and extracts have shown that the inhibition of amylase produces cytotoxic effects on the epithelial cells of the midgut of insects, which can cause mortality in larvae, in addition to decreasing the fertility and longevity of adult insects [11,69]. Studies with the essential oils of *Teucrium polium* and *Allium sativum* have shown inhibition of amylases due to destruction of the midgut epithelium and death of larvae of *Musca domestic* and *Ephestia kuehniella* Zeller, respectively [70,71]. Inhibition of amylases caused death in *Ae*. *aegypti* larvae, attributed to the destruction of enzyme-producing cells [47]. Thus, we suggest that the constituents present in the essential oil of *L*. *grata* act synergistically in a similar manner, causing destruction of the midgut epithelium and amylase-producing cells of *Aedes* spp., causing the death of the larvae. 

**Figure 8:**
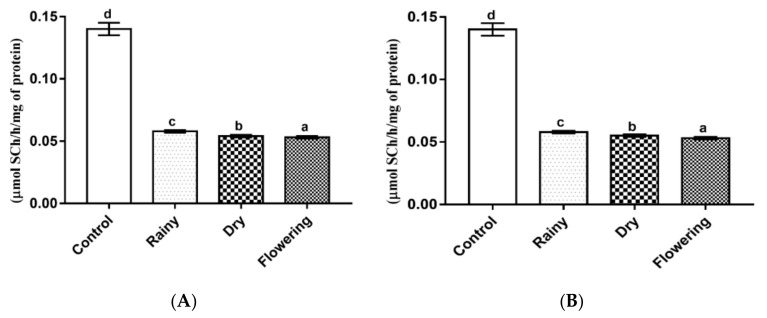
Biochemical changes in acetylcholinesterase of *Ae*. *aegypti* and *Ae*. *albopictus* larvae. Changes in acetylcholinesterase levels between *Ae*. *aegypti* (**A**) and *Ae*. *albopictus* (**B**) larvae treated and not treated with the essential oils of *Lippia grata*. Different lowercase letters in the bar denote significant difference (*p* < 0.05). Bars represent standard deviations (*n* = 3). Values estimated by one-way ANOVA followed by the Tukey test. Control, untreated larvae.

The *L. grata* essential oils promoted a significant reduction (*p* < 0.05) of acetylcholinesterase in the larvae. The Tukey test showed significant pairwise differences between the enzyme levels of the larvae treated with the three essential oils and of these in relation to the control (Figure 8A,B).

Acetylcholinesterase is essential for insects, since it acts to catalyze the hydrolysis of acetylcholine, preventing accumulation in the cholinergic site and consequent permanent stimulation of nerve fibers, causing paralysis and death. [72,73]. This enzyme is targeted by several insecticides, and the absence of its inhibition when using these products, may result from insects’ resistance to insecticides [74].

Castillo-Morales et al. [74] identified that a single type of essential oil can have multiple targets in insects, such as the essential oil of *Salvia officinalis*, which promoted DNA damage, altered mitochondrial bioenergetics, and inhibited AChE in *Ae*. *aegypti*, causing the vector to die. Other studies have identified that constituents of essential oils such as thymol and 1,8-cineole alone can alter the levels of esterases and phosphatases, as well as acetylcholinesterase in insects, acting as inhibitors or inactivators of metabolic responses, causing physiological changes and death [67,75,76]. Thus, acting synergistically or in isolation, chemical constituents can inhibit the enzyme AChE. The results obtained with the essential oils of *L*. *grata* from the three periods showed further inhibition of acetylcholinesterase in *Ae*. *aegypti* and *Ae*. *albopictus*. 

*L. grata* essential oil from dry period caused increases in acid phosphatase of *Ae*. *aegypti* larvae and esterases of *Ae*. *albopictus* larvae treated with REO and DEO, but the activity of the three oils against the enzymes of the vectors, inhibiting or increasing their production, showed that regardless the period of leaf collection, the chemical constituents present in the essential oil of *L*. *grata* are active against the enzymes of *Ae*. *aegypti* and *Ae*. *albopictus* and responsible for the death of the vectors.

Regarding species resistance, changes in enzyme levels and lethal concentrations may indicate the species with the best resistance to the essential oil constituents. The total proteins, alkaline phosphatase, amylases, and acetylcholinesterase were inhibited, the level of esterase increased, and lethal concentrations were higher in *Ae*. *albopictus*, while in *Ae*. *aegypti* total proteins, amylases, and acetylcholinesterase were inhibited, the level of acid phosphatase increased, and lethal concentrations were lower. Therefore, the increase in the level of esterases possibly provided better resistance to the constituents of the essential oils in *Ae*. *albopictus* despite the inhibition of the levels of most of the studied enzymes.

## 3. Materials and Methods

### 3.1. Plant Material

Three collections of the same population of *L*. *grata* were carried out during 2019. The collections took place in the rainy, flowering, and dry periods, in March, June, and September. All leaves were collected at 9:00 am in the Gadelha Mountains region (geographic coordinates, 6° 26′ 19” S and 39° 15′ 53” W), municipality of Iguatu, located in the interior of the state of Ceará, Brazil. The botanical identification was carried out at the Prisco Bezerra Herbarium of Federal University of Ceará, where a voucher specimen was deposited under number EAC 63412. 

### 3.2. Extraction of the Essential Oils

The essential oils of the leaves of *L*. *grata* from each period were extracted by hydrodistillation using a Clevenger apparatus. For this purpose, 600 g of leaves was used, which was placed in a 1 L round-bottom flask, along with distilled water until the sample was completely covered inside the flask. The procedure lasted approximately 3 h after the start of steam condensation, according to the method proposed by Teles et al. [77].

### 3.3. Chemical Analysis of the Essential Oils

The analysis of the chemical composition of the essential oils of the leaves of *L*. *grata* was carried out using the gas chromatography coupled to mass spectrometry(GC-MS) technique, and the resulting data were compared with the records in the NIST database and literature (retention time and ionic fragmentation). The quantification of the identified constituents was obtained based on the areas under the corresponding chromatographic peaks, using 10 mg of essential oil, previously diluted in 2.5 mL of chloroform. 

The GC-MS process was conducted with a Shimadzu QP-2010 chromatograph under the following conditions: Rtx-5MS column (5% diphenyl/95% dimethylpolysiloxane) with dimensions of 30 m × 0.25 mm × 0.25 µm df; He carrier gas (24.2 mL/min, in constant linear speed mode); initial temperature of 250 °C, in split mode (1:100); and detector temperature of 250 °C. The programmed column temperature was from 35 to 180 °C, at 4 °C/min, from 180 to 280 °C, at 17 °C/min, and then maintained at 280 °C for 10 min. Mass spectra were obtained at 70 eV electron impact. The sample was injected in a volume of 1 µL. Compounds were identified by the relative retention times for known compounds and by comparing them with the compounds present in the database of the National Institute of Standards and Technology (NIST) and in the published literature [27,78].

### 3.4. Larvicidal Activity

The larvicidal potential of essential oils from the leaves of *L*. *grata* was analyzed against third-instar larvae of *Ae*. *aegypti* and *Ae*. *albopictus*, collected from the colonies maintained in the laboratory, according to the parameters established by the World Health Organization (WHO) [79]. Concentrations of 100, 75, 50, 25, and 12.5 μg.mL^−1^ of the analyzed samples were prepared, pre-solubilized in 2.5% dimethyl sulfoxide (DMSO) and 97.5% distilled water, and a negative control was also tested with DMSO and water. Twenty larvae of each species were placed in 20 mL of each of solution. The bioassays were carried out in triplicate and lasted approximately 24 h. All experiments were accompanied by a control series, containing the same number of larvae in DMSO and distilled water. 

### 3.5. Activity of Essential Oils on Enzymes of Ae. aegypti and Ae. albopictus Larvae

For the enzymatic study, the live and dead third stage larvae treated at concentrations of 50 and 100 μg.mL^−1^ were separated, considering that compounds with LC_50_ values <100 μg.mL^−1^ had larvicidal effect [80]. The analyses were performed according to Suryawanshi et al. [56] and Ellman et al. [81], with modifications. The mechanism of action of the essential oils from the three periods (REO, FEO, and DEO) on the larvae of *Ae*. *aegypti* and *Ae*. *albopictus* was investigated from the analysis of the main enzymes (total proteins, alkaline phosphatase, acid phosphatase, proteases, esterases, amylases, and acetylcholinesterase) involved in detoxification and digestion of Culicidae larvae. Enzymatic tests were performed with a Bioplus Bio-200^®^ biochemical analyzer (Barueri, Brazil), for determination of total proteins, alkaline phosphatase, acid phosphatase, and amylase, using specific reagents (Labtest^®^), with standard samples, according to the manufacturer’s recommendations. Esterases and proteases were measured by radial immunodiffusion, with the aid of standard samples for the test. For acetylcholinesterase (AChE), the analyses were performed in triplicate, using a BioTek model ELX 800 ELISA reader (Vermont, USA), and the data were analyzed with the Gen5 V2.04.11 software. The analyses of the other enzymes were performed in triplicate using the standard reagent samples. The values of means and standard deviations of concentrations were plotted on graphs. To obtain the minimum volume for analysis, dilutions of the samples were used, maintaining the ideal concentrations for the test. Statistical analysis was performed using GraphPad Prism v5.01.

### 3.6. Statistical Analysis

Lethal concentrations (LC_50_ and LC_90_), confidence intervals, and comparison between essential oils were obtained through Probit analysis based on Finney’s probit model [82], Student’s t interval, and ANOVA with the Tukey multiple comparisons test using the statistical software R version 4.0.3 and Microsoft Excel 2016. To investigate significant differences in the enzymatic activities of larvae treated and not treated with the essential oil of *L*. *grata* (*p* < 0.05), ANOVA was used after confirming the normal distribution of the variables, followed by the Tukey test using the GraphPad Prism software v5.01.

## 4. Conclusions

The essential oils of *L*. *grata* leaves, collected in the three periods of the year were found to contain thymol and 1,8-cineole as the main constituents, differing from other oils of the same species collected in different regions of Northeast Brazil, suggesting a new chemotype from the Caatinga biome. The essential oils showed relevant larvicidal activity against both *Ae*. *aegypti* and *Ae*. *albopictus* by acting on important detoxification enzymes such as acetylcholinesterase, which was inhibited in both species, while alkaline phosphatase was inhibited in *Ae*. *Albopictus,* and esterases were increased in *Ae*. *aegypti*. The three oils had inhibitory action on the digestive enzyme amylases, preventing the nutritional processes, and possibly promoting death of the larvae of both species. The new chemotype was more active than other chemotypes previously described (<LC_50_), so it has promise to develop products to fight dengue vectors.

## Figures and Tables

**Table 1:** Chemical constituents of the essential oils of the leaves of *L*. *grata*.

Constituents	KI_Cal_	Content (%)		
		REO	DEO	FEO
Α-Thujene	926	0.19	−	−
α-Pinene	932	1.43	−	4.86
Sabinene	970	0.27	−	−
β-Pinene	972	0.26	−	−
Myrcene	988	1.74	−	1.32
α-Terpinene	1013	0.62	−	−
*p*-Cymene	1021	6.02	0.89	6.07
Limonene	1025	0.51	−	−
1,8-Cineole	1028	9.43	13.58	7.00
γ-Terpinene	1056	2.82	−	1.33
Terpinen-4-ol	1180	1.74	1.21	1.09
l-α-terpineol	1194	0.98	−	−
α-Terpineol	1195	1.32	2.66	1.38
Thymol methyl ether	1240	7.02	5.05	6.51
Thymol	1303	58.46	73.49	65.82
Carvacrol	1309	0.45	−	−
Thymol acetate	1357	1.42	3.12	3.58
α-Copaene	1376	0.69	−	−
*Z*-caryophyllene	1417	2.57	−	1.04
Delta-cadinene	1511	0.71	−	−
Caryophyllene oxide	1562	0.58	−	−
Total		99.23	100	100

REO, rainy season essential oil; DEO, dry season essential oil; FEO, flowering season essential oil. Kovats indexes (KI) were estimated by linear regression of retention times of the main compounds in the chromatograms and respective Kovats index from the literature [27].

**Table 2:** Main chemotypes of *Lippia grata* Schauer in the Brazilian States situated in Northeastern Brazil.

State	Chemotype/Percentage	Content (%)	Authors
Ceará	Thymol/carvacrol	44.4–22.2	Bitu et al., 2015 [25]
	Carvacrol/*p*-cymene	50.13–10.73	Neto et al., 2010 [31]
	Thymol/carvacrol	31–12	Santiago et al., 2005 [19]
Piauí	Carvacrol/*p*-cymene	48.12–24.39	Barriga et al., 2020 [28]
Paraiba	*p*-Cymene/carvacrol	22.2–20	Craveiro et al., 1981 [32]
Pernambuco	Carvacrol/thymol	76.8–6.98	Souza et al., 2017 [33]
Sergipe	Carvacrol/y-terpinene	53.77–9.37	Melo et al., 2019 [34]
	Thymol/methyl thymol	63.81–8.14	
Maranhão	Thymol/*p*-cymene	73.5–9.2	Franco et al., 2014 [35]

**Table 3:** Median lethal concentration of essential oil from *L*. *grata* leaves against third-instar larvae.

Oil	Larvae	LC_50_ ± SD (CI 95%)	LC_90_ ± SD (CI 95%)
**REO**	*Ae. Aegypti*	21.77 ^a^ ± 0.68 (20.09–23.45)	51.14 ^a^ ± 3.83 (41.62–60.66)
	*Ae. Albopictus*	35.99 ^c^ ± 0.54 (34.66–37.32)	67.91 ^dc^ ± 2.25 (62.32–73.5)
**DEO**	*Ae. Aegypti*	36.28 ^c^ ± 3.14 (28.48–44.08)	61.65 ^cb^ ± 1.30 (58.42–64.88)
	*Ae. Albopictus*	41.51 ^d^ ± 1.33 (38.21–44.81)	71.11 ^d^ ± 4.16 (60.77–81.45)
**FEO**	*Ae. Aegypti*	30.00 ^b^ ± 1.89 (25.3–34.7)	60.09 ^b^ ± 2.17 (54.69–65.49)
	*Ae. Albopictus*	46.06 ^d^ ± 0.80 (44.07–48.05)	89.29 ^e^ ± 1.66 (85.16–93.43)
**Control**	Vehicle (DMSO)	−	−

The control group did not show lethality against the larvae. The same superscript letters indicate statistical equality, according to the Tukey test. LC_50_, concentration that kills 50% of the exposed larvae (in μg.mL^−1^) with standard deviation (SD) and confidence interval of 95% (CI); LC_90_, concentration that kills 90% of the exposed larvae (in μg.mL^−1^) with standard deviation (SD) and confidence interval of 95% (CI). REO, rainy season essential oil; DEO, dry season essential oil; FEO, flowering season essential oil.

## Data Availability

The raw data of this research are with the corresponding authors and can be made available upon reasonable request.
